# 

**Published:** 1844-12

**Authors:** 


Vol. V. DECEMBER, 1844. No. II.

THE

AMERICAN

### JOURNAL AND LIBRARY

OF

### Dental Science.

PUBLISHED QUARTERLY.

PUBLISHED UNDER THE AUSPICES OF THE

**American Society of Dental Surgeons.**

EDITORS:

CHAPIN A. HARRIS M. D., D. D. S.

EDWARD MAYNARD, M. D., D. D. S.

AMOS WESTCOTT, M. D. D. D. S.

BALTIMORE:

ARMSTRONG & BERRY.

WOODS AND CRANE, PRINTERS.

$5 00 per annum, payable in advance.

This number contains 7 sheets.

